# Hypertensive Encephalopathy

**Published:** 2012

**Authors:** Mostafa Sharifian

**Affiliations:** Professor of Pediatric Nephrology, Pediatric Nephrology Research Center (PNRC), Shahid Beheshti University of Medical Sciences, Tehran, Iran

**Keywords:** Hypertension, Hypertensive encephalopathy, Children

## Objective

Hypertension is called the silent killer and vital organs such as the brain, eyes, kidneys and the heart are the targets. Seizure, central nervous system (CNS) hemorrhage, and cerebrovascular accident (CVA), blindness and heart attacks are the end points.

The prevalence of hypertension in children is much less than adults, but evidence reveals that the source of hypertension in adulthood goes back to childhood. In 70-80% of cases hypertension is due to renal diseases. In children, hypertensive encephalopathy (HE) may be the first manifestation of renal diseases. Seizure is one of the most common manifestations of HE.

In this article, definitions, etiology, pathophysiology and finally the acute and chronic managements of HE will be discussed.

## Introduction

Hypertension (HTN) is the second most common cause of death after diabetes in adults worldwide. Some authors call it the silent killer and others believe that it is not so silent (1, 2).

Between 1 and 5 percent of children and 15% of young adults suffer from hypertension while more than 60% of adults above the age of 65 years have hypertension. If the child’s blood pressure (BP) is more than the 90th percentile, the incidence of hypertension in adulthood rises 2-4 fold. Therefore, it is postulated that childhood diseases such as reflux nephropathy (RN) are the reason of hypertension in adulthood (3, 4).

Based on the World Health Organization (WHO) reports, hypertension is the cause of 62% of cerebrovascular accidents (CVA) and 49% of ischemic heart diseases. For every 5 mmHg increase in diastolic BP, there is a 35% and 20% increase in the risk of CVA and coronary artery disease, respectively. In addition, hypertension is the cause of up to 50% of end stage renal diseases (ESRD) requiring dialysis and transplantation in adults.

## Definitions

Since BP values differ with the child’s age, sex, height, weight and environment, definitions are based on the percentile of related monograms (3-7). If the BP is between the 90th and 95th percentile, the child is pre-hypertensive. Blood pressures between 95th and 99th percentile +5 mmHg are stage 1 and more than 99th percentile +5 mmHg are stage 2 hypertension (8, 9).

Accelerated HTN is defined when a BP which was mild or moderate suddenly rises. In this condition, there are some fundal changes, but no papilledema. When there is papilledema, the hypertension is called malignant (1-12).

In hypertensive encephalopathy with papilledema the patient has evidence of diffuse brain dysfunction such as severe headache, vomiting, blurred vision, seizure and coma. Seizure is the most common presenting sign, especially in infants and small children with hypertensive encephalopathy (HE) (1- 13).

## Pathophysiology

In prolonged hypertension, adaptive changes occur in arteries in order to prevent hyper-perfusion of the brain while maintaining its normal perfusion. On the other hand, when the blood pressure rises more than the regulatory threshold, vessel injury in the form of fibrinoid necrosis develops and ischemia and edema of the brain ensues. 

Vasoconstriction of the brain vessels which causes vascular permeability and brain edema and hemorrhage and ongoing signs and symptoms is the response to very high systemic BP. The above mentioned condition has been proposed by most authors. Increased intracranial pressure created by this process causes further increase in the systemic HTN by stretching the receptors in the floor of the fourth ventricle. This causes a vicious cycle leading to more severe brain injury, seizure, coma and death (8-12).

## Etiology

A wide variety of endocrine, neurologic, renal, cardiac disorders, drugs and intoxications may cause HTN and HE. Although essential hypertension is the cause of more than 90% of hypertensions in the general population and 80% of the causes in referral clinics in adults, in children under 6 years it is mostly secondary. Main causes of secondary hypertension are classified into four categories:

1- Renal or azotemic hypertension (80%) 

2- Rennin mediated hypertension (10-15%)

3- Mineralocorticoid induced hypertension (3-5%)

4- Catecholamine induced hypertension (2-5%)

**1-Renal HTN**

**Fig 1:**
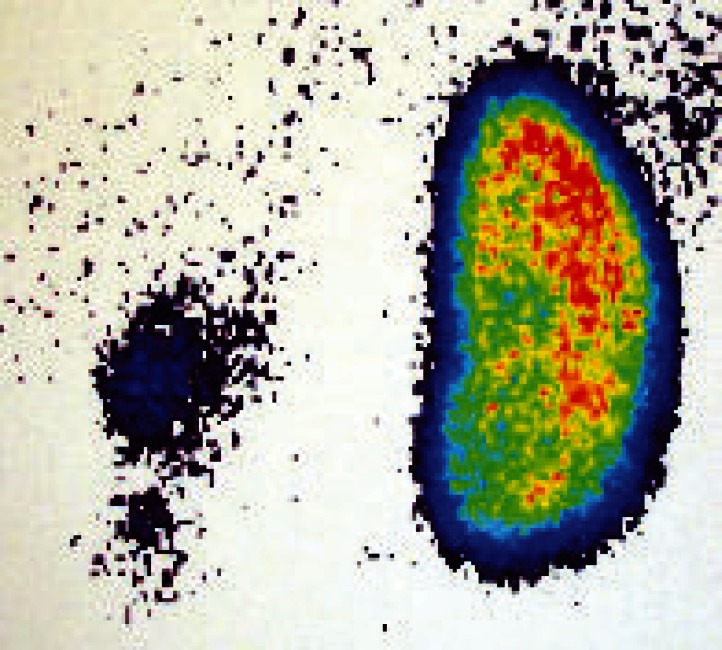
DMSA scan of a child with malignant hypertension showing a severe left renal scar

Patients with HTN due to renal disease usually have abnormal urinalysis (U/A), hematuria, proteinuria; high blood urea nitrogen (BUN); high blood creatinine (Cr); and they may have edema and oliguria. However, in 25% of the patients in this group, HTN is due to reflux nephropathy. Most of these patients have a normal BUN and Cr, but they have existing renal scars (Fig.1). 

Another important cause in this group is glomerulonephritis. If C3 is detected to be low, four groups of glomerulonephritides should be considered: 

post streptococcal glomerulonephritis (PSGN),

membranoproliferative glomerulonephritis (MPGN), 

lupus nephritis and finally glomerulonephritis of chronic infection. For accurate diagnosis these patients may need renal biopsy.

**2- Renin mediated HTN**

In this group, hypertension is caused by renovascular (stenosis in renal arteries) and cardiovascular (coarctation of the aorta) subgroups. These patients have four characteristics: 

a- They usually have hyperkalemic metabolic alkalosis, high rennin and aldosterone.

b- End organ damage such as proteinuria and left ventricular hypertrophy

c- Malignant hypertension is common and high diastolic pressure is significant.

d- Are refractory to usual antihypertensive medications and need specific medications such as Angiotensin Converting Enzyme Inhibitors (ACEI) for prompt control.

**3- Mineralocorticoid induced HTN**

These patients have hyperkalemic metabolic alkalosis and a high plasma aldosterone, but a low rennin. 

Diastolic blood pressure is mildly elevated and a prompt response to treatment with spironolactone is observed.

**4- Catecholamine induced HTN**

These patients usually present with malignant HTN and encephalopathy as they have very high systolic blood pressure readings up to 300 mmHg. They frequently follow head and neck surgeries and pheochromocytomas. 

The diagnosis is based on finding high fasting blood metanephrine and normetanephrine and vanylmandelic acid (VMA) and homovanilic acid (HVA) in the 24-hour urine and the source of the tumor can be detected by metaiodobenzylguanidine (MIBG) isotope scan (8-12).

Catecholamine-induced HTN can also be caused by Guillain Barre Syndrome (GBS), poliomyelitis and dysautonomia.

Severe hypertension has many other causes such as hyperthyroidism, Liddle’s syndrome and use of extra corporeal membrane oxygenation (ECMO). We ourselves presented a case of hypertensive encephalopathy due to mercury poisoning (14).

Malignant hypertension is rare in neonates and is mostly due to renal failure or congenital adrenal hyperplasia (CAH) (8-19).

In infancy, two-thirds of the causes are due to coarctation of the aorta and polycystic kidney diseases (PKD). 

Nephritis and hemolytic uremic syndrome (HUS) causes approximately 23% and the remaining causes include neuroblastoma or ganglioneuroblastoma, Wilm’s tumor, Cushing’s syndrome, pheochromocytoma, renal transplant rejection and drugs such as steroids.

**Clinical manifestations**

Patients with hypertension may present with fatigue, sleep disturbances and headache. Headache becomes more severe with higher blood pressures. Gradually, visual changes, chest pain and epistaxis ensue.

Congestive heart failure and infarction, pulmonary edema, dissecting aortic aneurism may accompany hypertensive encephalopathy which frequently manifests as generalized seizure in children.

More than 40% of malignant hypertensions develop HE, 20% of which were fatal in a study performed by Flynn and Tullus due to intracranial hemorrhage (20).

The earliest sign of HE includes irritability, lethargy, hypotonia and coma. After 12-36 hours of headache, generalized seizure occurs. Papilledema is present in one-third of the patients with HE.

Status epilepticus itself raises systolic and diastolic BP in 10% of the cases. Muscle twitching and myoclonus my occur progressing to focal neurologic signs such as aphasia scotomas and hemiparesis, brain stem failure and death (20). Furthermore, the child may have signs and symptoms of the underlying disease.

## Diagnosis of the cause of HTN

**History**

Family history: Presence of essential HTN and familial pheochromocytomas is worth considering and a history of umbilical catheterization in the neonatal period may lead to the diagnosis of renal artery stenosis and rennin mediated HTN. Presence of dysuria and frequency and a history of urinary tract infection (UTI) can lead to renal causes.

Drug history: Consumption of steroids, cyclosporine and ACTH should be sought. History of muscle cramps, weakness can lead to hyperaldosteronism.

Abdominal bruit in the physical examination may be a sign of renovascular HTN, webbed neck is a sign of turner syndrome and cafe au lait skin discoloration is a sign of neurofibromatosis.

**Screening tests**

Screening tests for evaluation of the cause of hypertension are summarized in Table 1.

**Table 1:** Screening Tests for Evaluation of the Cause of Hypertension (8- 10)

**Test**	**Findings**	**Significance**
CBC	Anemia Eosinophilia	CRF Interstitial Nephritis
U/A, U/C	Hematuria, proteinuria UTI	Glomerulonephritis Reflux Nephropathy
Electrolytes	Hypokalemic metabolic alkalosis	Rennin-Mediated HTN, Hyperaldosteronism
Lipid Profile, Uric Acid	Hyperlipidemia Hyperuricemia	Essential HTN

CBC: Complete Blood Count, U/A: Urinalysis, U/C: Urine Culture, CRF: Chronic Renal Failure, UTI: Urinary Tract Infection, HTN: Hypertension

In step 1, routine tests, electrolytes and ultrasound of kidneys and bladder are performed.

In step 2, dimercaptosuccinic acid scan (DMSA) is preformed instead of rapid sequence intravenous pyelography (RS-IVP), which is an old procedure (Fig. 1). Measurement of rennin, aldosterone, renal biopsy and angiography may be necessary. In step 3, renal vein rennin sampling in order to rule out renal artery stenosis and detection of the side (right or left kidney) that is more likely the cause of HTN.

## Management

For pharmacologic treatment of HTN there are more than nine groups of drugs (Table 2). Among them, direct vasodilators, alpha and beta blockers, calcium channel blockers and parenteral ACE inhibitors are the main groups used for the management of hypertensive emergencies and HE. 

Hypertension management is accomplished in two steps: 

1- Acute management

2- Chronic management

1- Acute management: when the patient is in stage 2 of HTN and in urgency or emergency HTN, decline of HTN should be stepwise as follows:

1-25% reduction in the first hour (safer to reduce 25% in the first 8 hours)

2-1/3 reduction in the first 6-8 hours

3-Not achieving below 95% before 24- 48 hours

Excessive reduction leads to diminished cerebral blood flow, syncope and infarction of the cerebral cortex, brainstem and retina.

Direct vasodilators commonly used for acute management are sodium nitroprusside, diazoxide and hydralazine, but labetolol (alpha and beta blocker) is the treatment of choice for our situation. The blood drug level should be monitored for nitroprusside as well as the cyanide and thiocyanate blood level and the infusion rate should be monitored regularly based on the intensive care unit (ICU) protocol. Hydralazine has a delayed starting effect and the peak level is reached in about 30 minutes which is very late especially for HE. Diazoxide and nifedipine have an unpredictable effect on the level of BP reduction. The mentioned items emphasize the reason why labetolol is the best choice in this situation. Drug dosages for hypertensive emergencies and HE are summarized in Table 3.

**Table 2:** Drug Categories for Pharmacologic Treatment of Hypertension

Diuretics Beta Blockers ACE Inhibitors Angiotensin Receptor Blockers Calcium Channel Blockers Alpha 1 Blockers Centrally Acting Alpha 2 Agonists Direct Vasodilators Peripherally Acting Adrenergic Antagonists

**Table 3:** Management of Hypertensive Encephalopathy in Children (1-14)

	Drug		Dose
Acute management	Direct vasodilators	Nitroprusside	0.3-8µg/kg/min
		Diazoxide	1-3 mg/kg IV over 5-10 min
		Hydralazine	0.1-0.3 mg/kg IV, repeated every 4-6 hr
	Calcium Chanel Blocker	Nicardipine	0.5-5µg/kg/min
	β blocker	Esmolol	100-500µg/kg IV then 50-500 µg/kg/min
	α and β blocker	Labetolol	0.2-1 mg/kg/dose IV over 2 min repeat every 5-10 min, max dose 60 mg
	α blocker	Phentolamine (For Cathecolamine- induced HTN)	0.1-0.2 mg/kg/IV bolus (max 5mg) Repeated every 2-4 hr May be given 1-2 hr before surgery
Chronic management	ACE inhibitors	Captopril	0.3-0.5 mg/kg/dose (tid) Maximum: 6 mg/kg/ day
		Enalapril	0.08 mg/kg/day up to 40 mg/day
		Lisinopril	0.07 mg/kg/day up to 40 mg/day
	ARBs	Losartan	0.7 mg/kg /day up to 100 mg/day
		Irbesartan	75-150 mg/day
	Adrenergic clocker	Atenolol	Initial: 0.5-2 mg/kg/day
		Propranolol	1-2 mg/kg /day (bid-tid) Maximum: 4 mg/kg/day
	Calcium Chanel blocker	Nifedipine	0.25-0.5 mg/kg /day Maximum: 3 mg/kg/day
		Amlodipine	0.06 mg/kg/day
	Direct vasodilators	Minoxidil	0.1-0.2 mg/kg/day (Maximum: 50 mg/day)
	Diuretics	Furosemide	0.5-4.0 mg/kg/dose Maximum: 6 mg/kg/day
		Hydrochlorothiazide	1 mg/kg per day (q.d.; bid) Maximum: 3 mg/kg /day
		Spironolactone	1 mg/kg /day (q.d.-bid) Maximum: 3.3 mg/ day

For HE due to catecholamine induced HTN, phentolamine and phenoxybenzamine are the drugs of choice.

When HTN came below 95% the patient should not be left alone as the BP goes up again; but second line drugs such as hydralazine, nifedipine, atenolol, captopril and other ACE inhibitors, diuretics and angiotensin receptor blockers (ARBs) are continued base on the etiology of HTN.

**Posterior reversible encephalopathy syndrome (PRES)**

**Fig 2:**
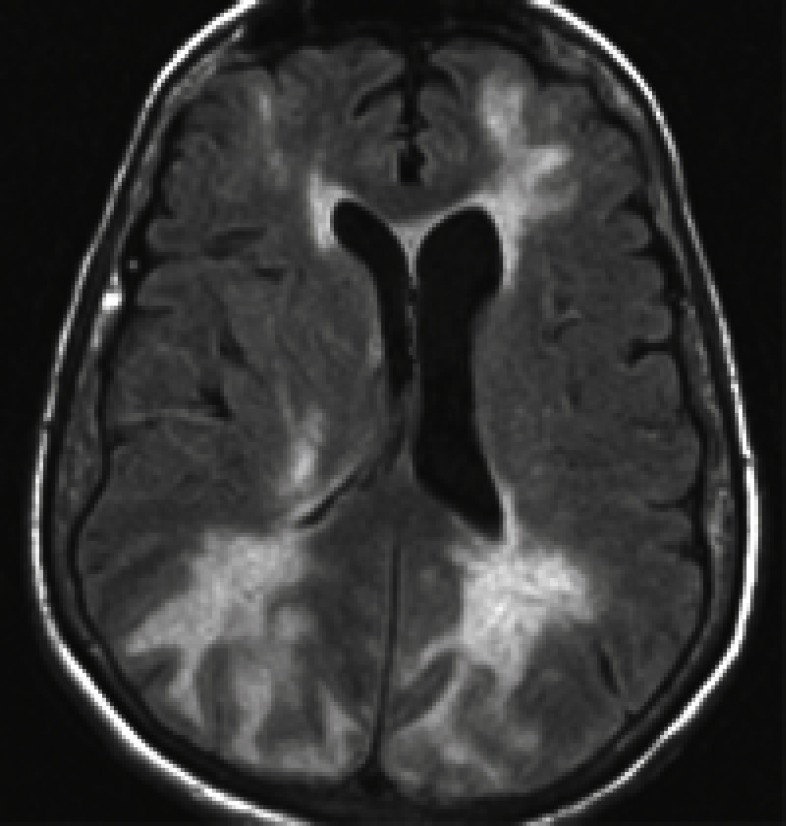
Brain MRI in a patient with posterior reversible encephalopathy syndrome (PRES) shows multiple areas of hyperintense signal

Occipital blindness, headache, lethargy, transient motor deficit, confusion, visual hallucination generalized convulsion are defined as posterior reversible leukoencephalopathy or posterior reversible encephalopathy syndrome (PRES) another manifestation of the (18-21). This entity may also be caused by drugs such as acyclovir and cyclosporine. MRI is the imaging of choice. In T1-weighted images, dark abnormalities and in T2-weighted images, bright abnormalities which are due to edema are seen (Fig. 2) (21). Multiple infarctions are sometimes noted in HE. In the EEG of these cases, the rhythm is slow.

Treatment consists of 30˚ head elevation, intracranial pressure (ICP) monitoring and reduction of edema with osmotic agents. In newborns, the treatment is started with nifedipine (15, 19).
